# DKK1 inhibits breast cancer cell migration and invasion through suppression of β-catenin/MMP7 signaling pathway

**DOI:** 10.1186/s12935-019-0883-1

**Published:** 2019-06-24

**Authors:** Jie Niu, Xiao-Meng Li, Xiao Wang, Chao Liang, Yi-Dan Zhang, Hai-Ying Li, Fan-Ye Liu, Hua Sun, Song-Qiang Xie, Dong Fang

**Affiliations:** 1 Institute for Innovative Drug Design and Evaluation, School of Pharmacy, Henan University, N. Jinming Ave, Kaifeng, 475004 China; 2 Institute of Chemical Biology, School of Pharmacy, Henan University, N. Jinming Ave, Kaifeng, 475004 China

**Keywords:** DKK1, β-Catenin, MMP7, Breast cancer, Migration and invasion

## Abstract

**Background:**

DKK1 has been reported to act as a tumor suppressor in breast cancer. However, the mechanism of DKK1 inhibits breast cancer migration and invasion was still unclear.

**Methods:**

Western blot and real time PCR was used to detect the expression of DKK1, β-catenin and MMP7 in breast cancer cells. Wound scratch assay and transwell assay was employed to examine migration and invasion of breast cancer cell.

**Results:**

DKK1 overexpression dramatically inhibits breast cancer cell migration and invasion. Knockdown of DKK1 promotes migration and invasion of breast cancer cells. DKK1 suppressed breast cancer cell migration and invasion through suppression of β-catenin and MMP7 expression. XAV-939, an inhibitor of β-catenin accumulation could reverse DKK1 silencing-induced MMP7 expression in breast cancer cells. Meanwhile, XAV-939 also could reverse the increase in the cell number invaded through Matrigel when DKK1 was knockdown. Furthermore, depletion of MMP7 also could reverse DKK1 knockdown-induced increase in the cell number invaded through Matrigel.

**Conclusions:**

DKK1 inhibits migration and invasion of breast cancer cell through suppression of β-catenin/MMP7 pathway, our findings offered a potential alternative for breast cancer prevention and treatment.

## Background

Dickkopf-1 (DKK1), a secreted protein, was first found in Xenopus and involved in the head formation and limb morphogenesis during vertebrate development [1, 2]. Abnormal expression of DKK1 has now emerged as an important regulator in a variety of human cancers [3, 4]. For example, some reports discovered that DKK1 was overexpressed in hepatocellular carcinoma (HCC) and myeloma, acting as a tumor promoter [5, 6]. By contrast, DKK1 expression was downregulated in renal cell carcinoma and colorectal cancers, indicating that it might function as a tumor suppressor [7, 8].

The significance of DKK1 expression in breast cancer progression and prognosis remains largely unknown. Some studies have suggested that DKK1 acts as a putative tumor suppressor in breast cancer [9, 10]. However, the mechanism of DKK1 inhibits breast cancer metastasis was still unclear. Matrix metalloproteinase-7 (MMP-7), a secreted zinc- and calcium-dependent endopeptidase, is one of the most important target genes downstream of Wnt/β-catenin signaling [11, 12]. Its expression was associated with poor prognosis in tumors of the pancreas, colon, brain and breast cancers [13–15]. To our knowledge, it remains unclear whether DKK1 inhibits breast cancer metastasis through suppression of MMP-7 expression. In this study, our results suggested DKK1 inhibited migration and invasion by suppression of β-catenin expression which in turn downregulates the expression of matrix metalloproteinase 7 (MMP7).

## Materials and methods

### Materials

RPMI 1640 and fetal bovine serum (FBS) were purchased from Gibco (Grand Island, NY, USA). Antibodies against DKK1 (ab109416), β-catenin (ab32572) and MMP7 (ab5706) were purchased from Abcam (Cambridge, MA, USA). β-actin antibody (sc-47778), goat anti-rabbit IgG-HRP (sc-2004) and goat anti-mouse IgG-HRP (sc-2005) were purchased from Santa Cruz Biotechnology, Inc. (Dallas, TX, USA). MMP7 siRNA (sc-41553) was obtained from Santa Cruz Biotechnology (Santa Cruz, CA, USA).

### Cell lines and cell culture

All the breast cancer cell lines used in this study were purchased from the cell bank of the Chinese Academy of Science (Shanghai, China) and cultured in RPMI 1640 medium supplemented with 10% fetal bovine serum. The cells were maintained at 37 °C in a 5% CO_2_ humidified incubator.

### Plasmid construction and transfection

DKK1 overexpression and shRNA expression plasmids were constructed as previous described [16]. Briefly, full-length coding region of DKK1 was amplified from human genomic DNA by reverse transcription-polymerase chain reaction (RT-PCR). Then the PCR products were digested with *Xho*I/*Bam*HI and were inserted into the pIRES2-EGFP vector. The recombinant construct was verified by direct DNA sequencing. For the construction of shRNA expression plasmids, a shRNA sequence targeted human DKK1 transcript (accession no. NM_012242.2; sense 5′-GGA ATAAGTACCAGACCATTG-3′) was selected for RNA interference (RNAi). A scrambled sequence (sense 5-GGAATAAGACCATGACCATTG-3′) was used as a negative control.

Transfections of plasmids into breast cancer cells were carried out using Lipofectamine^®^2000 reagent (Invitrogen, Thermo Fisher Scientific, Inc., USA) according to the manufacturer’s instructions. Briefly, plasmids and transfection reagent were each diluted with RPMI 1640 medium, mixed together, and incubated for 20 min at room temperature. Then the mixture was added to the medium for transfection. At 4 h post-transfection, the cell culture medium was replaced with RPMI 1640 medium supplemented with 10% fetal bovine serum. RNA interference was performed using Lipofectamine^®^ RNAiMAX (Life Technologies) according to the manufacturer’s instructions. After 24 h, these transfected cells were collected to perform the following experiments.

### mRNA extraction and real-time PCR analysis

Total RNA of breast cancer cells was isolated using TRIzol (Invitrogen, Carlsbad, CA, USA) according to the manufacturer’s instructions. Then, 1 µg DNase-treated RNA was reverse transcribed to cDNA using random hexamers and MMLV reverse transcriptase according to the manufacturer’s instructions (Takara, Tokyo, Japan). Relative quantitative real-time PCR was performed with SYBR Premix Ex Taq II (Takara). The reaction conditions were as follows: 95 °C, pre-denaturation for 10 min, 15 s at 95 °C, and 1 min at 60 °C for a total of 40 cycles. Glyceraldehyde phosphate dehydrogenase (GAPDH) was selected as an internal control. The relative expression level of the genes was calculated by the 2^−ΔΔ^Ct method. The following primers were used for real-time PCR reactions: DKK1, forward: 5′-CTG CAG TCA GGA CTC TGG GA-3′, reverse: 5′-AAC TAT CAC AGC CTA AAG GGA A-3′; MMP7, forward: 5′-TGT ATG GGG AAC TGC TGA CA-3′, reverse: 5′-GCG TTC ATC CTC ATC GAA GT-3′; GAPDH, forward: 5′-GAC ACC CAC TCC TCC ACC TTT-3′, reverse: 5′-TTG CTG TAG CCA AAT TCG TTG T-3′.

### Wound scratch assay

The wound scratch assay was performed as previously described [17]. Cells were seeded in a 24-well plate and cultured, after cells had grown to confluence, a wound was scratched with a plastic tip and incubated for 24 h in RPMI 1640 medium. Photographs were taken all along the wound at the beginning and at the end of the experiment.

### Cell invasion assay

The invasion assays were performed as previously described [18]. Briefly, 2 × 10^5^ cells were seeded in the top of 8 μm chamber coated with a Matrigel (Corning, Bedford, MA, USA), the lower chamber was filled with 600 μL RPMI 1640 supplemented with 10% FBS. The inhibitor used in this study was added to both the top and bottom chambers of the Transwell. After incubation for 24 h, cells were fixed with 4% paraformaldehyde, stained with 0.1% crystal violet. The number of invading cells was determined by counting randomly on each membrane.

### Western blot analysis

Cell lysates were extracted using RIPA buffer (20 mM Tris–HCl pH 7.5, 2 mM EDTA, 150 mM NaCl, 1 mM sodium vanadate, 10 mM NaF, 2.5 mM sodium pyrophosphate, 1% sodium deoxycholate, 0.1% SDS, 1% NP-40) supplemented with a protease inhibitor cocktail (Roche, Mannheim, Germany). The nuclear protein was isolated using the nuclear protein and cytoplasmic protein extraction kit (Beyotime, Shanghai, China) following the manufacturer’s instruction. The concentration of protein was measured with a BCA assay kit (Thermo Scientific, IL, USA). Equal amounts of protein samples (20 µg) were separated through SDS-polyacrylamide gel electrophoresis using 10% running gels and transferred onto PVDF membranes (Bio-Rad Laboratories, CA, USA). After blocking with 5% non-fat dry milk, the PVDF membranes were incubated with the indicated primary and secondary antibodies and detected by using the ECL plus reagents (Beyotime, Shanghai, China). The western blots were visualized using a FluroChem E Imager (Protein Simple, Santa Clara, CA, USA). Quality One Software was used to calculate the alteration of corresponding protein expression.

### Statistical analysis

Statistical analyses were performed using the GraphPad Prism 5.0 (GraphPad Software, Inc., La Jolla, CA, USA). All data are presented as means ± SEM. The difference between the two groups was tested by Student's test. One-way analysis of variance (ANOVA) followed by Tukey or Dunnet’s post-tests was used to compare means of multiple experimental groups. Differences with p < 0.05 were considered statistically significant.

## Results

### DKK1 inhibits breast cancer cells migration and invasion in vitro

**Fig. 1:**
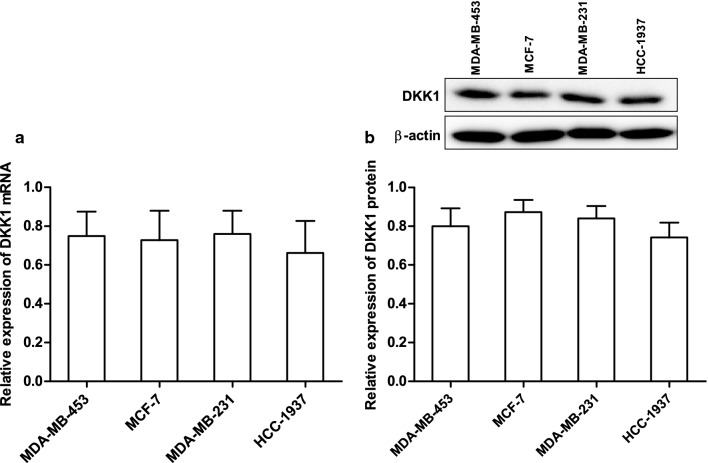
The level of DKK1 mRNA and protein expression was detected in breast cancer cells. **a** Real time PCR assay suggested the expression of DKK1 mRNA was in a similar degree in the four breast carcinoma cell lines. **b** Western blot assay suggested the expression of DKK1 protein was in a similar degree in the four breast carcinoma cell lines

**Fig. 2:**
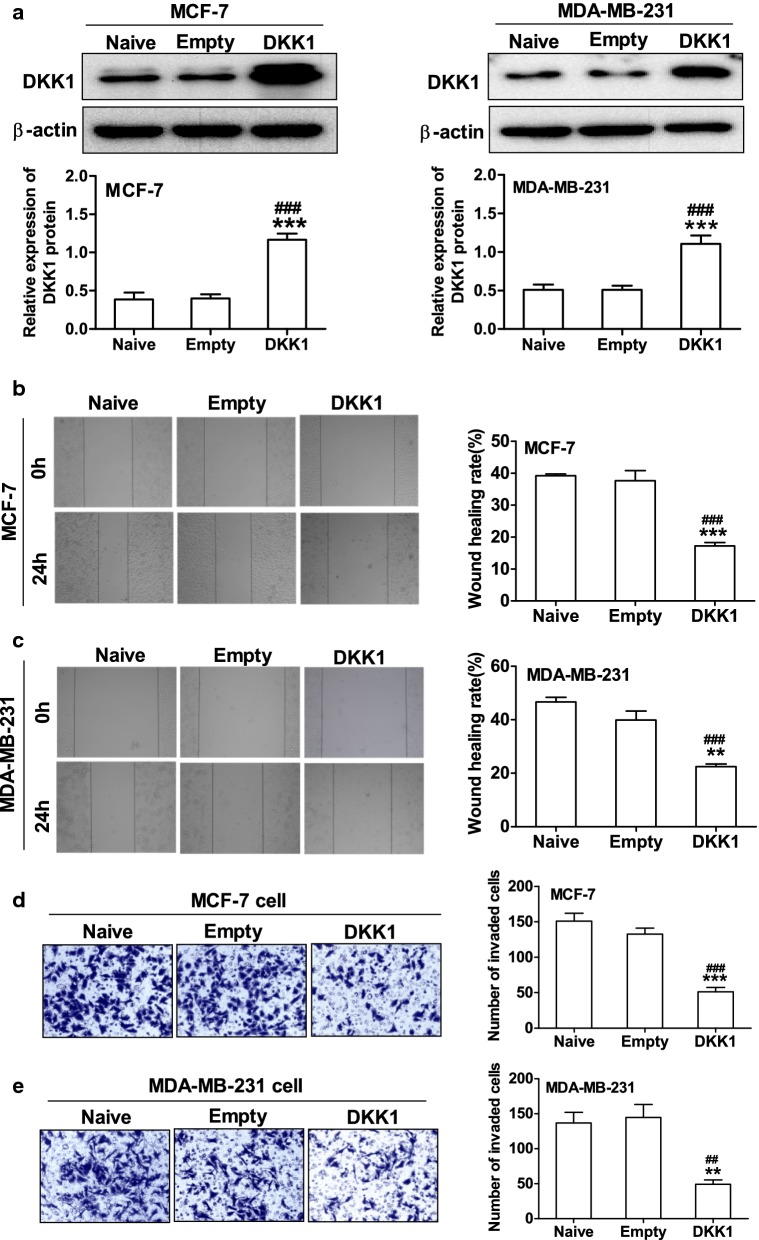
DKK1 overexpression attenuated the ability of migration and invasion in MCF-7 and MDA-MB-231 cells. **a** Western blot of DKK1 in MCF-7 and MDA-MB-231 cells after transfected with empty vector or pIRES2-DKK1 plasmid. Note that DKK1 was overexpressed in MCF-7 and MDA-MB-231 cells after transfected pIRES2-DKK1 plasmid. Results are presented as mean ± SEM, ***p < 0.001 compared to the empty vector group, ^###^p < 0.001 compared to the naive group, n = 5 per group, one-way ANOVA. Naïve: untreated group, Empty: empty vector group, DKK1: DKK1 overexpressed group. Wound scratch assay showed overexpression of DKK1 attenuated cell migration in MCF-7 (**b**) and MDA-MB-231 (**c**) cells. Left: representative images of wound scratch. Right: histograms represent the analysis of the wound healing rate. Results are presented as mean ± SEM, **p < 0.01, ***p < 0.001 compared to the empty vector group, ^###^p < 0.001 compared to the naive group, n = 5 per group, one-way ANOVA. Naïve: untreated group, Empty: empty vector group, DKK1: DKK1 overexpressed group. Transwell chamber coated with Matrigel assay suggested overexpression of DKK1 attenuated the cell number invaded through Matrigel in MCF-7 (**d**) and MDA-MB-231 cells (**e**). Left: representative images of transwell assay. Right: histograms represent the analysis of the number of invaded cells. Results are presented as mean ± SEM, **p < 0.01, ***p < 0.001 compared to the empty vector group, ^##^p < 0.01, ^###^p < 0.001 compared to the naive group, n = 5 per group, one-way ANOVA. Naïve: untreated group, Empty: empty vector group, DKK1: DKK1 overexpressed group

**Fig. 3:**
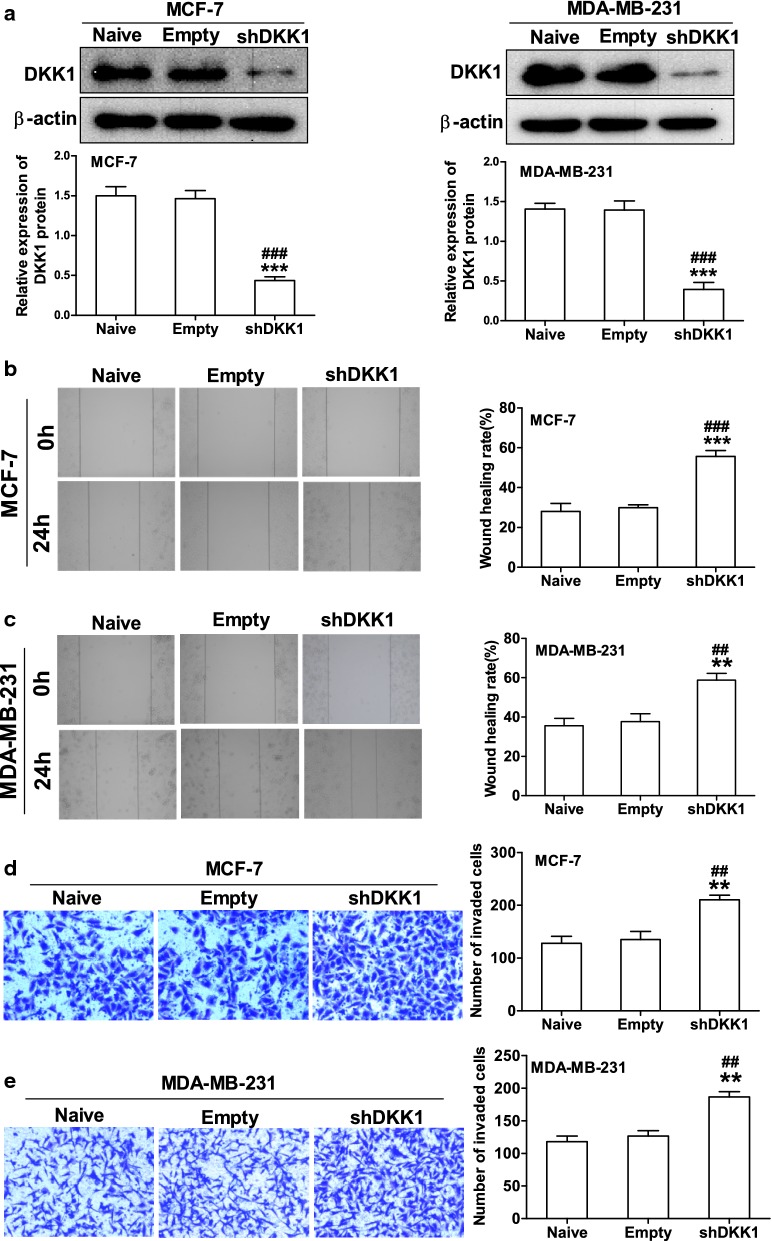
Knockdown of DKK1 enhanced the ability of migration and invasion in MCF-7 and MDA-MB-231 cells. **a** Western blot of DKK1 protein expression in MCF-7 and MDA-MB-231 cells after transfected with empty vector or DKK1 shRNA. Note that DKK1 was knocked down in MCF-7 and MDA-MB-231 cells after transfected DKK1 shRNA. ***p < 0.001 compared to the empty vector group, ^###^p < 0.001 compared to the naive group, n = 5 per group, one-way ANOVA. Naïve: untreated group, Empty: empty vector group, shDKK1: DKK1 knockdown group. Wound scratch assay showed knockdown of DKK1 enhanced cell migration in MCF-7 (**b**) and MDA-MB-231 (**c**) cells. Left: representative images of wound scratch. Right: histograms represent the analysis of the wound healing rate. Results are presented as mean ± SEM, **p < 0.01, ***p < 0.001 compared to the empty vector group, ^##^p < 0.01, ^###^p < 0.001, n = 5 per group, one-way ANOVA. Naïve: untreated group, Empty: empty vector group, shDKK1: DKK1 knockdown group. Transwell chamber coated with Matrigel assay suggested knockdown of DKK1 increased the cell number invaded through Matrigel in MCF-7 (**d**) and MDA-MB-231 cells (**e**). Left: representative images of transwell assay. Right: histograms represent the analysis of the number of invaded cells. Results are presented as mean ± SEM, **p < 0.01 compared to the empty vector group, ^##^p < 0.01 compared to the naive group, n = 5 per group, one-way ANOVA. Naïve: untreated group, Empty: empty vector group, shDKK1: DKK1 knockdown group

To determine whether DKK1 could regulate breast cancer cells migration and invasion, we first examined DKK1 expression in 4 different breast carcinoma cell lines (MDA-MB-453, MCF-7, MDA-MB-231 and HCC-1937) using real time PCR and Western blot. As the results shown in Fig. 1, the mRNA and protein expression of DKK1 was in a similar degree in these breast carcinoma cell lines. Then MCF-7 and MDA-MB-231 cell lines were selected and transfected pIRES2-DKK1 plasmid to set up overexpression of DKK1. Furthermore, we also used shRNA to knock down DKK1 expression in MCF-7 or MDA-MB-231 cells. As Fig. 2 shown, the cells transfected with DKK1 displayed a decreased ability in wound closure compared with the cells transfected with empty vector in MCF-7 and MDA-MB-231 cells. Next, to determine the role of DKK1 in tumor cell invasion, we performed the experiments using transwell chamber with Matrigel. As illustrated in Fig. 2, overexpression of DKK1 in MCF-7 and MDA-MB-231 cells significantly attenuated the cell numbers that invaded through Matrigel in comparison with the cells transfected with empty vector. In contrast, knockdown of DKK1 in MCF-7 and MDA-MB-231 cells enhanced cells migration and invasion using wound scratch assay and transwell assay (Fig. 3). These results indicated that DKK1 play an inhibitory role in breast cancer cells migration and invasion in vitro.

### DKK1 downregulates the expression of MMP7 via the repression of Wnt/β-catenin signaling pathway in breast cancer

**Fig. 4:**
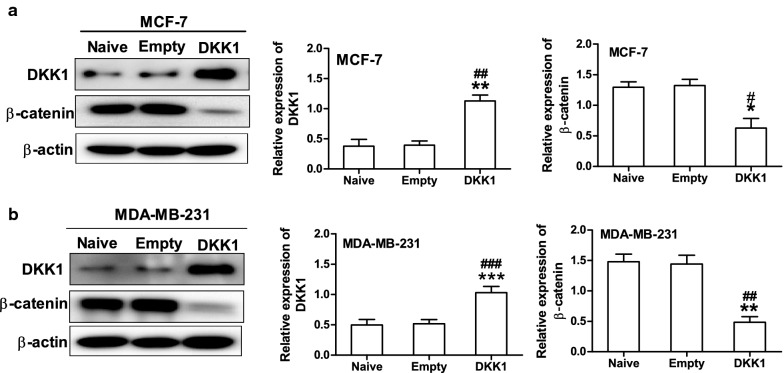
Overexpression of DKK1 inhibited β-catenin protein expression in MCF-7 (**a**) and MDA-MB-231 (**b**) cells. β-actin was used as an internal reference of total protein, results are presented as mean ± SEM, *p < 0.05, **p < 0.01, ***p < 0.001 compared to the empty vector group, ^#^p < 0.05, ^##^p < 0.01, ^###^p < 0.001 compared to the naive group, n = 5 per group, one-way ANOVA. Naïve: untreated group, Empty: empty vector group, DKK1: DKK1 overexpressed group

**Fig. 5:**
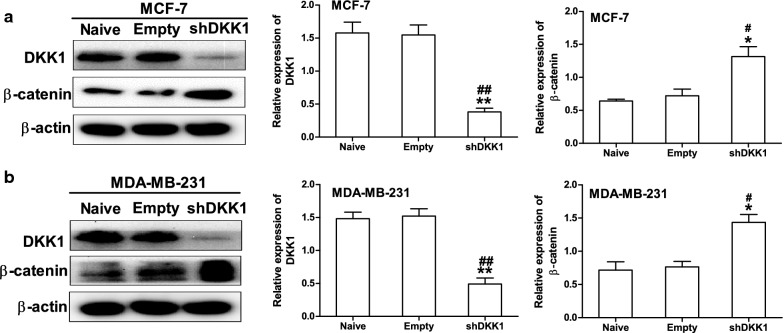
Knockdown of DKK1 promoted β-catenin protein expression in MCF-7 (**a**) and MDA-MB-231 (**b**) cells. β-actin was used as an internal reference of total protein, results are presented as mean ± SEM, *p < 0.05, **p < 0.01 compared to the empty vector group, ^#^p < 0.05, ^##^p < 0.01 compared to the naive group, n = 5 per group, one-way ANOVA. Naïve: untreated group, Empty: empty vector group, shDKK1: DKK1 knockdown group

DKK-1 has been demonstrated as an inhibitor of Wnt/β-catenin signaling pathway which plays a pivotal role in tumorigenesis [3, 19]. Therefore, we asked whether DKK1 suppressed cell migration and invasion by regulating β-catenin expression in breast cancer cells. Through Western blot, we found the expression of β-catenin was downregulated in MCF-7 and MDA-MB-231 cells transfected with DKK1 (Fig. 4). On the other hand, knock down of DKK1 promoted β-catenin expression in MCF-7 and MDA-MB-231 cells (Fig. 5). Collectively, these data demonstrated that β-catenin was an important downstream target of DKK1.

**Fig. 6:**
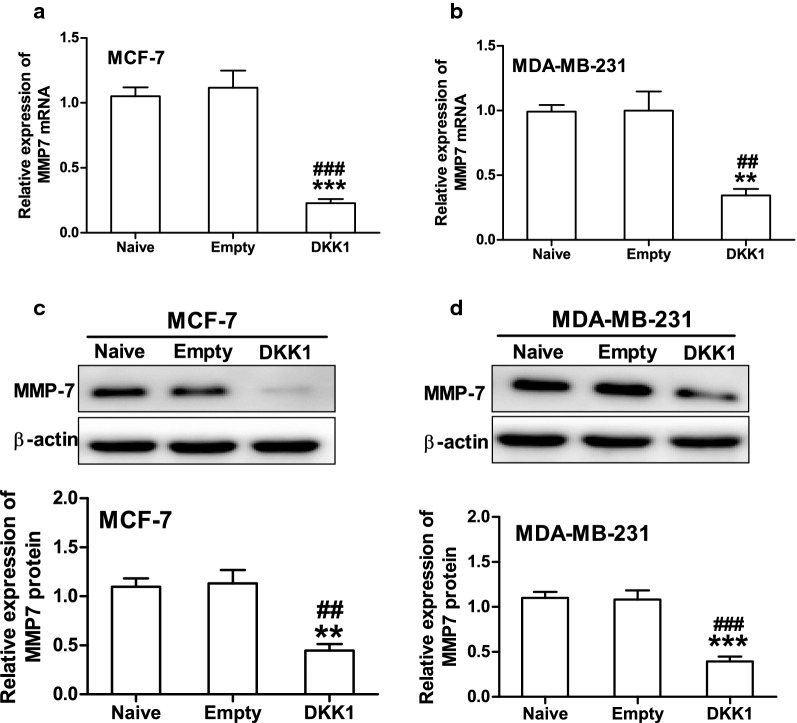
Overexpression of DKK1 inhibited the mRNA and protein expression of MMP7 in MCF-7 and MDA-MB-231 cells. Real time PCR analysis suggested overexpression of DKK1 inhibited the mRNA expression of MMP7 in MCF-7 (**a**) and MDA-MB-231 (**b**) cells. Results are presented as mean ± SEM, **p < 0.01, ***p < 0.001 compared to the empty vector group, ^##^p < 0.01, ^###^p < 0.001 compared to the naive group, n = 5 per group, one-way ANOVA. Naïve: untreated group, Empty: empty vector group, DKK1: DKK1 overexpressed group. Western blot assay showed overexpression of DKK1 inhibited the protein expression of MMP7 in MCF-7 (**c**) and MDA-MB-231 (**d**) cells. Results are presented as mean ± SEM, **p < 0.01, ***p < 0.001 compared to the empty vector group, ^##^p < 0.01, ^###^p < 0.001 compared to the naive group, n = 5 per group, one-way ANOVA. Naïve: untreated group, Empty: empty vector group, DKK1: DKK1 overexpressed group

**Fig. 7:**
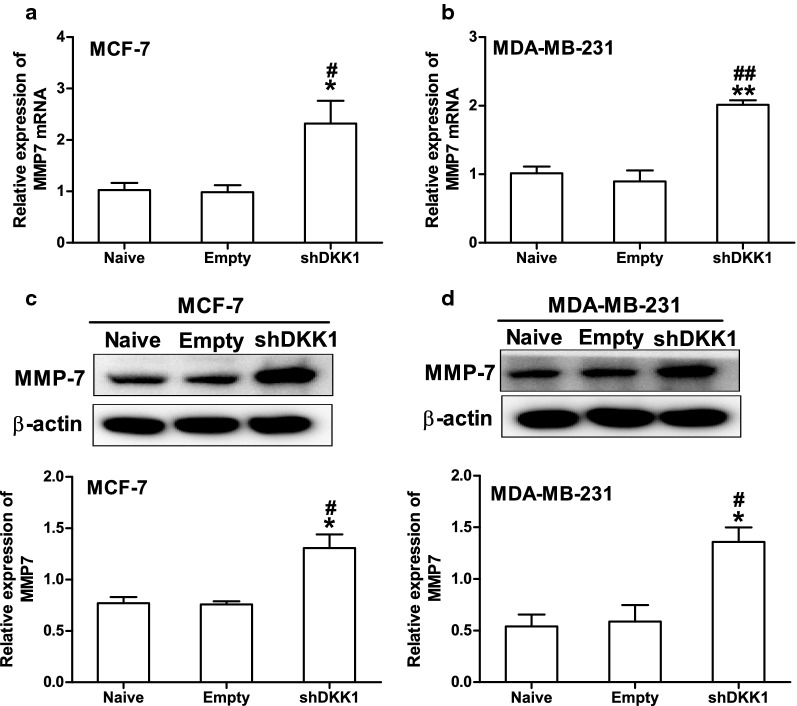
Knockdown of DKK1 promoted the mRNA and protein expression of MMP7 in MCF-7 and MDA-MB-231 cells. Real time PCR analysis suggested overexpression of DKK1 enhanced the mRNA expression of MMP7 in MCF-7 (**a**) and MDA-MB-231 (**b**) cells. Results are presented as mean ± SEM, *p < 0.05, **p < 0.01 compared to the empty vector group, ^#^p < 0.05, ^##^p < 0.01 compared to the naive group, n = 5 per group, one-way ANOVA. Naïve: untreated group, Empty: empty vector group, shDKK1: DKK1 knockdown group. Western blot assay showed overexpression of DKK1 inhibited the protein expression of MMP7 in MCF-7 (**c**) and MDA-MB-231 (**d**) cells. Results are presented as mean ± SEM, *p < 0.05 compared to the empty vector group, ^#^p < 0.05 compared to the naive group, n = 5 per group, one-way ANOVA. Naïve: untreated group, Empty: empty vector group, shDKK1: DKK1 knockdown group

**Fig. 8:**
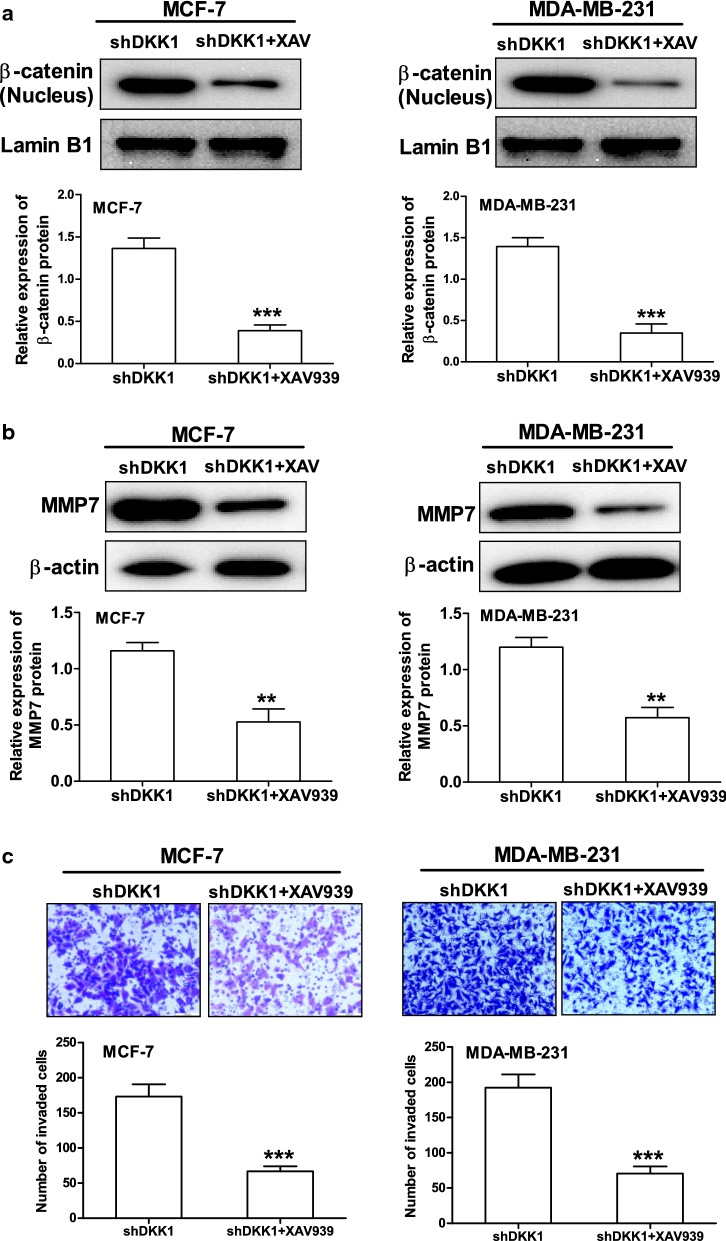
The inhibitor of β-catenin accumulation XAV-939 could reverse DKK1 knockdown-induced MMP7 protein expression and cell invasion in MCF-7 and MDA-MB-231 cells. **a** Western blot assay suggested XAV-939 could inhibit β-catenin nuclear accumulation in MCF-7 and MDA-MB-231 cells treated with DKK1 shRNA. Lamin B1 is used as an internal control. Results are presented as mean ± SEM, ***p < 0.001, n = 5 per group, Student's test was used. **b** Western blot assay suggested XAV-939 could reverse DKK1 knockdown-induced MMP7 protein expression in MCF-7 and MDA-MB-231 cells. Results are presented as mean ± SEM, **p < 0.01, n = 5 per group, Student's test was used. **c** Transwell chamber coated with Matrigel assay showed XAV-939 could reverse DKK1 knockdown-induced the cell invasion in MCF-7 and MDA-MB-231 cells. Results are presented as mean ± SEM, ***p < 0.001, n = 5 per group, Student's test was used

**Fig. 9:**
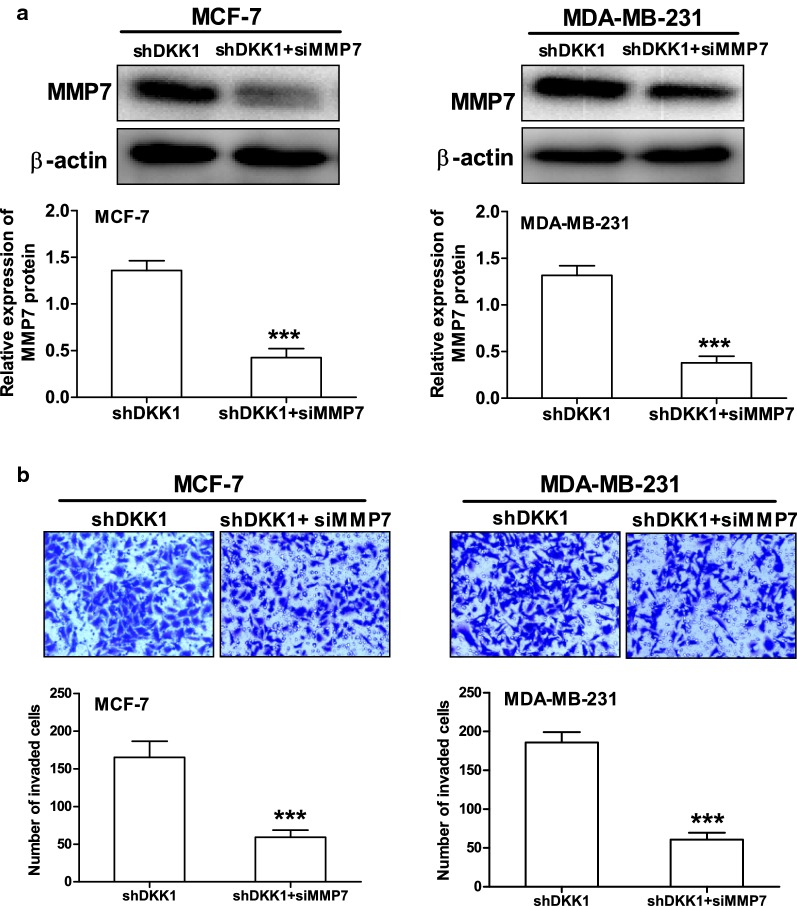
Knockdown of MMP7 with specific siRNA could reverse DKK1 knockdown-induced the cell invasion in MCF-7 and MDA-MB-231 cells. **a** Western blot assay suggested MMP7 siRNA could attenuate the protein expression of MMP7 in MCF-7 and MDA-MB-231 cells treated with DKK1 shRNA. Results are presented as mean ± SEM, ***p < 0.001, n = 5 per group, Student's test was used. **b** Transwell chamber coated with Matrigel assay showed MMP7 siRNA could reverse DKK1 knockdown-induced the cell invasion in MCF-7 and MDA-MB-231 cells. Results are presented as mean ± SEM, ***p < 0.001, n = 5 per group, Student's test was used

MMP7 is one of the target genes downstream of β-catenin/TCF signaling and plays a crucial role in promoting tumor cell migration and invasion [11, 12]. Therefore, we then investigate whether DKK1 regulates the expression of MMP7 in breast cancer cells. As expected, DKK1 overexpression significantly decreased the expression of MMP7 mRNA and protein in MCF-7 and MDA-MB-231 cells (Fig. 6). Vice versa, when DKK1 was knocked down, the mRNA and protein expression of MMP7 was dramatically increased in MCF-7 and MDA-MB-231 cells (Fig. 7). In order to confirm that MMP7 was one of the dominant downstream effector of β-catenin, we explored whether the effect of XAV-939, an inhibitor of β-catenin accumulation could reverse MMP7 expression induced by DKK1 knockdown. Using Western blot, we found that DKK1 knockdown-induced the upregulation of MMP7 was blocked by XAV-939 in MCF-7 and MDA-MB-231 cells (Fig. 8). Subsequently, we also found XAV-939 could reverse DKK1 knockdown-induced increase in the cell number invaded through Matrigel (Fig. 8). Furthermore, depletion of MMP7 also could reverse DKK1 knockdown-induced increase in the cell number invaded through Matrigel (Fig. 9). Taken together, these results showed that DKK1 inhibits breast cancer migration and invasion through suppression of β-catenin/MMP7 signaling pathway.

## Discussion

Dickkopf-1 (DKK1), a secreted protein, plays different roles in different types of cancers [20, 21]. For instance, DKK1 was overexpressed in pancreas carcinoma and non-small cell lung cancer where it promotes cancer cells to migrate, invade and proliferate [22, 23]. A downregulation of DKK1 has been observed in melanoma and colon cancer where it functioned as a tumor suppressor gene [24, 25]. In this study, our results showed DKK1 overexpression resulted in decreased cell migration and invasion in breast cancer cells. Consistent with our results, reports from other labs also suggested DKK1 could inhibit migration and invasion of breast cancer cells [26, 27]. However, emerging evidences found breast cancer-derived DKK1 inhibits osteoblast differentiation and osteoprotegerin expression which is associated with the presence of bone metastases [19, 28]. These contradicting observations may be due to the differences in tumor stage or tissue origin.

The Wnt/β-catenin signaling pathway has been widely implicated as a controller of cell growth, migration and stem-like phenotype [29, 30]. In the present study, we found introduction of DKK1 into breast cancer cells inhibited β-catenin protein expression in breast cancer cells, while knockdown of DKK1 promote β-catenin expression. In support of our findings, Koch et al. [31] reported that the downregulation of DKK1 increased proliferation of epithelial cells in the large intestine, which was associated with increased transcriptional activity of β-catenin. While Zhou et al. [32] suggested DKK1 overexpression inhibits proliferation and migration in human retinal pigment epithelial cells via the Wnt/β-catenin signaling pathway.

Matrix metalloproteinase-7 (MMP-7) is a small secreted proteolytic enzyme with broad substrate specificity [33]. Its expression is associated with tumor invasion, metastasis and survival in a variety of cancers [34, 35]. Several reports have shown that MMP7 is overexpressed in both the cells and tissues of breast cancer [36, 37]. Moreover, it is well-known that MMP7 is one of the most important downstream target genes of β-catenin/TCF-4 [11]. However, the relationship between MMP7 and DKK1 remain elusive in breast cancer. Here, we demonstrate MMP7 is an important target downstream of DKK1 in breast cancer cells, based on the following studies: knockdown of DKK1 is associated with enhanced level of MMP7 in MDA-MB-231 and MCF-7 cells. Overexpression of DKK1 represses MMP7 expression and inhibits migration and invasion of breast cancer cells. Moreover, upon treatment with β-catenin nuclear translocation inhibitor XAV-939 in MCF-7 and MDA-MB-231 cells, the protein level of MMP7 was reduced associated with a less invasive activity. All these findings suggested that DKK1 may inhibit breast cell migration and invasion by attenuating β-catenin/MMP7 signaling pathway. However, accumulating evidences suggested the famous downstream target genes of β-catenin including c-myc and MMP9 were remarkably correlated with breast cancer distant metastasis and poor prognosis [38, 39]. Therefore, we did not exclude these β-catenin target genes involved in the invasion process.

## Conclusion

DKK1 suppressed breast cancer cell migration and invasion by alleviating β-catenin/MMP7, our findings offered a potential alternative for breast cancer prevention and treatment.

## Data Availability

All data supporting the findings of this study are included in this published article.

## Abbreviations

DKK1: Dickkopf-1
HCC: hepatocellular carcinoma
MMP7: matrix metalloproteinase-7
GAPDH: glyceraldehyde phosphate dehydrogenase
